# Establishment and validation of in-house cryopreserved CAR/TCR-T cell flow cytometry quality control

**DOI:** 10.1186/s12967-021-03193-7

**Published:** 2021-12-24

**Authors:** Yihua Cai, Michaela Prochazkova, Chunjie Jiang, Hannah W. Song, Jianjian Jin, Larry Moses, Nikolaos Gkitsas, Robert P. Somerville, Steven L. Highfill, Sandhya Panch, David F. Stroncek, Ping Jin

**Affiliations:** Department of Transfusion Medicine and Cellular Engineering, Center for Cellular Engineering, NIH Clinical Center, NIH, 10 Center Drive-MSC-1184, Building 10, Room 3C720, Bethesda, MD 20892-1184 USA

**Keywords:** Multiparametric flow cytometry-based assay, Quality control, Chimeric antigen receptor (CAR) T-cells, T-cell receptor (TCR)-engineered T-cells, Cellular cancer immunotherapy

## Abstract

**Background:**

Chimeric antigen receptor (CAR) or T-cell receptor (TCR) engineered T-cell therapy has recently emerged as a promising adoptive immunotherapy approach for the treatment of hematologic malignancies and solid tumors. Multiparametric flow cytometry-based assays play a critical role in monitoring cellular manufacturing steps. Since manufacturing CAR/TCR T-cell products must be in compliance with current good manufacturing practices (cGMP), a standard or quality control for flow cytometry assays should be used to ensure the accuracy of flow cytometry results, but none is currently commercially available. Therefore, we established a procedure to generate an in-house cryopreserved CAR/TCR T-cell products for use as a flow cytometry quality control and validated their use.

**Methods:**

Two CAR T-cell products: CD19/CD22 bispecific CAR T-cells and FGFR4 CAR T-cells and one TCR-engineered T-cell product: KK-LC-1 TCR T-cells were manufactured in Center for Cellular Engineering (CCE), NIH Clinical Center. The products were divided in aliquots, cryopreserved and stored in the liquid nitrogen. The cryopreserved flow cytometry quality controls were tested in flow cytometry assays which measured post-thaw viability, CD3, CD4 and CD8 frequencies as well as the transduction efficiency and vector identity. The long-term stability and shelf-life of cryopreserved quality control cells were evaluated. In addition, the sensitivity as well as the precision assay were also assessed on the cryopreserved quality control cells.

**Results:**

After thawing, the viability of the cryopreserved CAR/TCR T-cell controls was found to be greater than 50%. The expression of transduction efficiency and vector identity markers by the cryopreserved control cells were stable for at least 1 year; with post-thaw values falling within ± 20% range of the values measured at time of cryopreservation. After thawing and storage at room temperature, the stability of these cryopreserved cells lasted at least 6 h. In addition, our cryopreserved CAR/TCR-T cell quality controls showed a strong correlation between transduction efficiency expression and dilution factors. Furthermore, the results of flow cytometric analysis of the cryopreserved cells among different laboratory technicians and different flow cytometry instruments were comparable, highlighting the reproducibility and reliability of these quality control cells.

**Conclusion:**

We developed and validated a feasible and reliable procedure to establish a bank of cryopreserved CAR/TCR T-cells for use as flow cytometry quality controls, which can serve as a quality control standard for in-process and lot-release testing of CAR/TCR T-cell products.

**Supplementary Information:**

The online version contains supplementary material available at 10.1186/s12967-021-03193-7.

## Introduction

T-cells genetically engineered to express chimeric antigen receptors (CAR) or T-cell receptors (TCR) have emerged as a promising immunotherapy approach for the treatment of hematologic malignancies and some types of solid tumors [1, 2]. Since the first anti-CD19 CAR T-cell product was approved for the treatment of acute lymphoblastic leukemia (ALL) by the US Food and Drug Administration (FDA) in 2017, several more CAR T-cell products targeting CD19 and other antigens have been developed [3, 4]. Currently, more than six hundred CAR/TCR T-cell clinical trials are underway.

As living drugs, clinical-grade CAR/TCR T-cell products are manufactured in compliance with current good manufacturing practices (cGMP) [5]. Ensuring the quality of CAR/TCR T-cell products is essential in each step of manufacturing process, including the flow cytometry-based assays used for quality assurance assessment of the CAR/TCR T-cells [6–8].

Flow cytometry-based assays are robust, efficient and powerful tools for the analysis, characterization and evaluation of cells during the CAR/TCR T-cell manufacturing process [9]. Manufacturing CAR/TCR T-cells is a complex process involving blood collection followed by T-cell selection, activation, vector transduction and expansion [7, 10]. During this process, multiparametric flow cytometry-based evaluation plays an important role in monitoring the manufacturing steps including in-process and lot-release product testing [9]. The percentage of cells expressing CD3 (T-cell), CD4/CD8 T-cell subset composition and transduction efficiency are almost always measured using flow cytometry. Since some flow cytometry reagents used to assess transduction efficiency react with multiple types of CAR or TCR T-cells, laboratories manufacturing multiple genetically engineered T-cell products may need to include an assay in the lot-release testing process that ensures the correct vector was used during the manufacturing process [7, 9]. This identity testing may be performed with molecular assays, but it is often preformed using flow cytometry [8, 11]. All these parameters are considered critical criteria for evaluating the quality of clinical CAR/TCR T-cells before infusing them into the patient.

The wide variation in the type of samples analyzed and the subjective nature of flow cytometry assays make it important to establish a standard or quality control in order to evaluate the results and to monitor the entire process including sample preparation, reagent quality, staining and instrument performance [12, 13]. More importantly, a standard or quality control also ensures the accuracy of flow cytometry assays, which is especially critical for the CAR/TCR T-cell final product lot-release testing [14]. Off-the-shelf flow cytometry standards are available for some applications such as lymphocyte immunophenotyping [15]. These standards can be used to monitor a portion of the in-process and lot-release flow cytometry testing of CAR/TCR-T cell products. However, a standard for CAR/TCR T-cell product flow cytometry evaluation is not commercially available, partially due to the large variety of CAR/TCR T-cell products being manufactured.

Our institution is a GMP-compliant facility which manufactures CAR/TCR T-cell products for phase I/II clinical trials, thus it is essential for us to establish procedures to generate in-house cryopreserved transduced CAR/TCR T-cell final products for use as flow cytometry quality controls and validate these controls. To this end, we cryopreserved aliquots from two representative CAR-T cell products and one TCR-engineered T-cell product. The long-term stability of cryopreserved CAR/TCR T-cell control cells as well as their post-thaw shelf-life were evaluated by measuring transduction efficiency and in addition, for one control cell, vector identity analysis was performed. The sensitivity of flow cytometric analysis using the cryopreserved quality controls was also determined by evaluating serial dilutions of cryopreserved control cells with untransduced cells. Finally, we compared the precision of analysis of these cryopreserved quality controls among different technicians and different instruments. Thus, the in-house cryopreserved CAR/TCR T-cell flow cytometry quality control was established and validated. The validation schema was shown in the Additional file 1: Fig. S1.

## Materials and methods

### CAR/TCR T-cell manufacturing

Two CAR T-cell products (CD19/CD22 bispecific CAR T-cells and FGFR4 CAR T-cells) and one TCR engineered T-cell product (KK-LC-1 TCR T-cells) were manufactured in Center for Cellular Engineering (CCE), NIH Clinical Center using their clinical cell therapy manufacturing protocols. In brief, for CD19/CD22 bispecific CAR T-cells and FGFR4 CAR T-cells, autologous peripheral blood mononuclear cells (PBMCs) collected by apheresis were subjected to anti-CD4/CD8 double-positive selection for T-cell enrichment, followed by lentivirus transduction and expansion in an automated instrument (CliniMACS Prodigy, Miltenyi Biotech). The CAR T-cells were harvested at day 9 and cryopreserved. For KK-LC-1 TCR engineered T-cells, an autologous PBMCs product was collected by apheresis and transfected with a retroviral vector that encoded a TCR recognizing KK-LC-1. The cells were expanded and harvested at day15 and cryopreserved.

### Cryopreservation and thaw

Final CAR/TCR-T cell products were cryopreserved by using coolCell^®^ alcohol-free freezing containers with CS10 (Biolife solutions) and 4% human serum albumin (HSA) Plasma-Lyte A at 1:1 ratio as a cryoprotectant. The cells were cryopreserved in vials each of which contained approximately 5–10 million cells. The cryopreserved vials were stored in a liquid nitrogen tank. The cells were thawed using a ThawSTAR CFT2 (Biolife solutions) and resuspended in HBSS after removing the cryoprotectant by centrifugation. The concentration of viable cells post-thaw was measured by Cellometer AUTO 2000 (Nexcelom Bioscience) using Acridine Orange/Propidium iodide (AO/PI) staining.

### Flow cytometric analysis

For flow cytometric analysis, one million cells were used for staining. The samples were stained with fluorochrome-labeled anti-human CD3, CD4, CD8 and CD45 antibodies (Abs) as well as viability dye 7-AAD (BD Biosciences) to assess viability and CD4/CD8 T cell frequencies. In addition, for CD19/CD22 bispecific CAR T-cells, samples were stained with purified protein-L (ThermoFisher Scientific), CD22-Fc (recombinant human Siglec-2, R&D systems) and CD19-Fc (CD19 CAR detection reagent, human, Biotin, Miltenyi Biotec) to determine the transduction efficiency and identity followed by corresponding secondary Abs: fluorochrome-labeled anti-protein L secondary Ab (ThermoFisher Scientific), anti-human IgG secondary Ab or streptavidin. For FGFR4 CAR T-cells and KK-LC-1 TCR T-cells, samples were stained with fluorochrome-labeled anti-human EGFR Ab (BioLegend) or anti-mouse TCR beta Ab (ThermoFisher Scientific) to determine the transduction efficiency of FGFR4 CAR T-cells and KK-LC-1 TCR T-cells, respectively. After 20 min of incubation at 4 °C, the samples were washed on BD FACS™ Lyse Wash Assistant (LWA), then acquired on BD FACSCanto II and BD FACSCanto 10-Color (BD Biosciences). A total of thirty thousand events were collected for each sample. Data were analyzed with BD FACSDiva™ software and FlowJo software (BD Biosciences). In brief, the viable CD3^+^ cell population was gated from the 7-AAD^−^ population of singlets, and then CD4^+^/CD8^+^ cells as well as cells expressing transduction efficiency and/or identity markers were further analyzed. The fluorescence minus one (FMO) controls for each transduction efficiency and identity marker were used to determine the positive gate for transduction efficiency and identity marker analysis on thawed quality control cells.

### Long-term stability of the cryopreserved CAR/TCR T-cell flow cytometry quality control cells

Cryopreserved CAR/TCR T-cell flow cytometry quality control cells were thawed after storage in liquid nitrogen for various periods of time (2 weeks, 1 month, 2 months, 3 months, 6 months, 9 months and 12 months). A minimum two vials of cryopreserved quality control cells were thawed at each time point. Samples were stained immediately with surface markers as well as viability dye 7-AAD. The viability, CD3 percentage, CD4/CD8 composition and transduction efficiency and identity were examined by flow cytometry.

### Stability of the thawed CAR/TCR T-cell flow cytometry quality control cells

Cryopreserved CAR/TCR T-cell flow cytometry quality control cells were thawed and resuspended in HBSS after centrifuging to remove the cryoprotectant. The cells were stored at room temperature and stained at 0 h, 2 h, 4 h and 6 h after thawing. The viability, CD3 percentage, CD4/CD8 composition and transduction efficiency and identity were examined by flow cytometry. Two independent experiments were conducted to determine the post-thaw shelf-life of quality control cells.

### Assessment of the sensitivity of flow cytometry assays using CAR/TCR T-cell flow cytometry quality control cells

Cryopreserved CAR/TCR T-cell flow cytometry quality control cells were thawed and mixed with their corresponding untransduced cells in serial dilutions (1:1, 1:2, 1:4, 1:8, 1:16 and 1:32). The transduction efficiency and identity were assessed by flow cytometry to determine the lowest limit of quantification and linearity of expression. Two independent experiments were conducted to assess the sensitivity of flow cytometry assays using these quality control cells.

### Assessment of the precision of flow cytometry assays using CAR/TCR T-cell flow cytometry quality control cells

Cryopreserved CAR/TCR T-cell flow cytometry quality controls were thawed and stained by two laboratory staff or one stained sample was measured and analyzed on two flow cytometry instruments (BD FACSCanto II and BD FACSCanto 10-Color). The precision assays between technicians and instruments were conducted at all time points across the one-year long-term stability study as described above. The expression of the transduction efficiency and identity markers were determined and compared by flow cytometry.

### Statistical analysis

All quantitative data are shown as mean ± standard deviation (SD) unless otherwise indicated. Coefficient of variance (CV) was also calculated and listed in the supplemental tables. The sensitivity study was performed by Pearson correlation coefficient statistics. A p value less than 0.05 was considered significant. Statistical analysis was performed with GraphPad Prism software and R package.

## Results

### Generation of CAR/TCR T-cell flow cytometry quality control cells

Two CAR T-cell products: CD19/CD22 bispecific CAR T-cells and FGFR4 CAR T-cells and one TCR-engineered T-cell product: KK-LC-1 TCR T-cells were manufactured in CCE, NIH Clinical Center. The cells were harvested at the end of culture and their characteristics were evaluated by flow cytometry in triplicates, including the viability, percentages of CD3^+^, CD4^+^ and CD8^+^ T-cell composition, as well as the transduction efficiency and/or identity expression. The gating strategy for Protein L expression on CD19/CD22 bispecific CAR T-cells was shown in the Additional file 1: Fig. S2 as an example, in which Protein L^+^ cell population was gated from viable CD3^+^ cells.

**Fig. 1:**
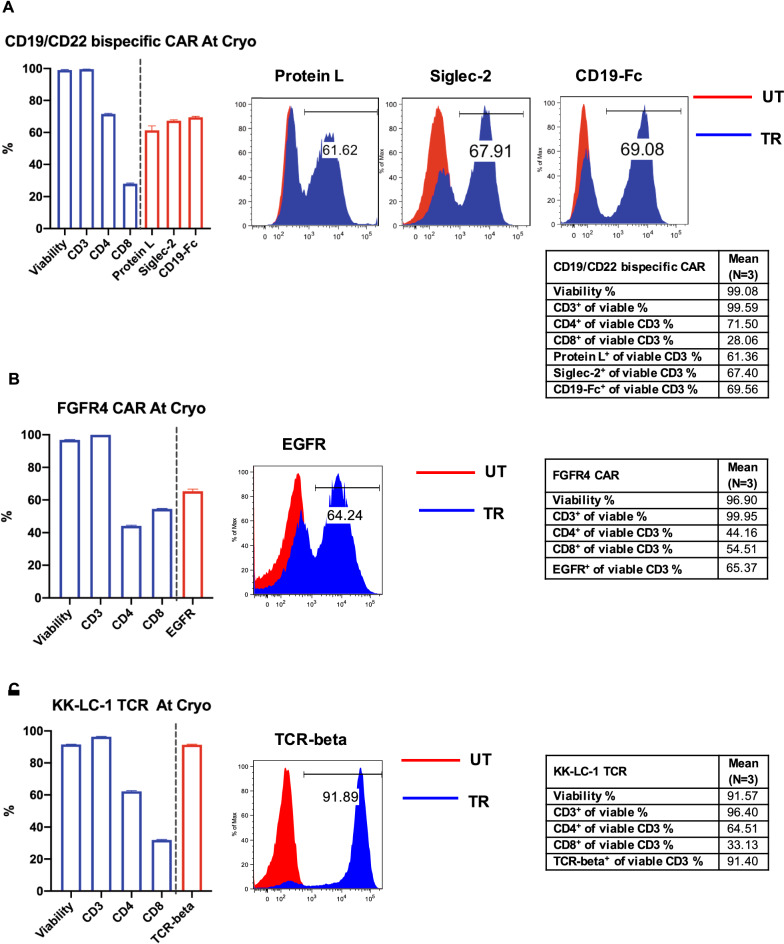
Generation of CAR/TCR T-cell flow cytometry quality control cells. Cells from two CAR T-cell products: CD19/CD22 bispecific CAR T-cell (**A**) and FGFR4 CAR T-cell (**B**) and one TCR engineered T-cell product: KK-LC-1 TCR T-cell (**C**) were harvested and stained. Flow cytometry was used to measure cell viability; CD3, CD4 and CD8 frequencies; and the expression of CAR/TCR T-cell markers: protein L, CD22-Fc (Siglec-2) and CD19-Fc for CD19/CD22 bispecific CAR T-cell, EGFR for FGFR4 CAR T-cell and murine TCR-beta for KK-LC-1 TCR T-cell. Data are shown as mean ± SD. The representative flow cytometry histograms were gated on viable CD3^+^ cells showing the results of analysis for protein L, CD22-Fc (Siglec-2), CD19-Fc (**A**), for EGFR (**B**) and for murine TCR-beta (**C**). Results from analysis of untransduced (UT) cells are shown in red and transduced (TR) cells are shown in blue in flow cytometry histogram. The mean values of each parameter for three quality control cells are summarized in each table

The viability of the fresh control cells was assessed by flow cytometric analysis via 7-AAD staining. As shown in Fig. 1A–C, the viability of the fresh CAR/TCR T-cell quality control cells was over 90%, which was consistent with AO/PI viability measurement via Cellometer AUTO 2000 (data not shown). As expected, there were mostly CD3^+^ cells, and of those CD3^+^ T cells, mostly CD4^+^ and CD8^+^ cells.

For CD19/CD22 bispecific CAR T-cells, the transduction efficiency of viable CD3^+^ T-cells was assessed by measuring the expression of protein L and the identity of the CAR T-cells was measured by the expression of CD22-Fc (Siglec-2) as well as CD19-Fc. Protein L is an immunoglobulin (Ig)-binding protein that binds to the variable light chains (kappa chain) of Ig. It is a general marker for the detection of CAR expression and often used to determine the transduction efficiency of CAR-T cell product [16]. For CAR-T cell identity detection, the specific markers targeting individual CAR-T cell are employed in assays. In this study, the percentage of CD19/CD22 bispecific CAR T-cells binding with protein L was 61.36% ± 2.80%. The percentages of the bispecific CAR T-cells reacting with CD22-Fc (Siglec-2) and CD19-Fc were 67.40% ± 0.60% and 69.56% ± 0.46%, respectively (Fig. 1A). Since FGFR4 CAR T-cells contains a truncated EGFR (EGFRt) within the construct, EGFR expression was utilized to determine the transduction efficiency. The percentage viable CD3^+^ T cells among the FGFR4 CAR T-cells expressing EGFR was 65.37% ± 1.27% (Fig. 1B). For the KK-LC-1 TCR T-cell product, the expression of murine TCR-beta was used to assess the transduction efficiency. The percentage of viable CD3^+^ T-cells expressing murine TCR-beta was 91.40% ± 0.37% (Fig. 1C). The characteristics of each CAR/TCR T-cell flow cytometry quality control at cryopreservation were summarized in the Additional file 2: Table S1.

The CAR/TCR T-cells were cryopreserved and stored in liquid nitrogen. These cryopreserved cells were evaluated for use as in-house CAR/TCR T-cell flow cytometry quality controls.

### Evaluation of the long-term stability of the cryopreserved CAR/TCR T-cell flow cytometry quality control cells (closed-vial stability)

Long-term stability is a critical quality characteristic of the quality control cells. The long-term stability of the cryopreserved flow cytometry assay quality control cells was determined by the measurement of the capability of the cells to generate reproducible and consistent flow cytometric analysis results over long periods of storage. Although it is well known that cryopreserved cells stored in liquid nitrogen can be maintained for many years [17], there are few reports about the life-span of cryopreserved CAR T-cell products nor the stability of expression of their cell surface markers. Therefore, we sought to determine the long-term stability of our cryopreserved CAR/TCR T-cell controls in flow cytometry assays. The criterion we used in the study for flow cytometry assay stability was a comparison of parameters measured by flow cytometry after thawing with those measured at time of cryopreservation. According to the current standard operating procedure (SOP) for the post-thaw quality control (PTQC) in our institution, if the values obtained from thawed cells fell within ± 20% range of the values measured at cryopreservation, the cells were considered to be stable. The one hundred percentage was a cut-off value if the upper bound (value at cryopreservation (Value Cryo) + Value Cryo X 20%) exceeded 100. In addition, the viability of thawed cells was required to be over 50% as measured by flow cytometric analysis via 7-AAD staining.

**Fig. 2:**
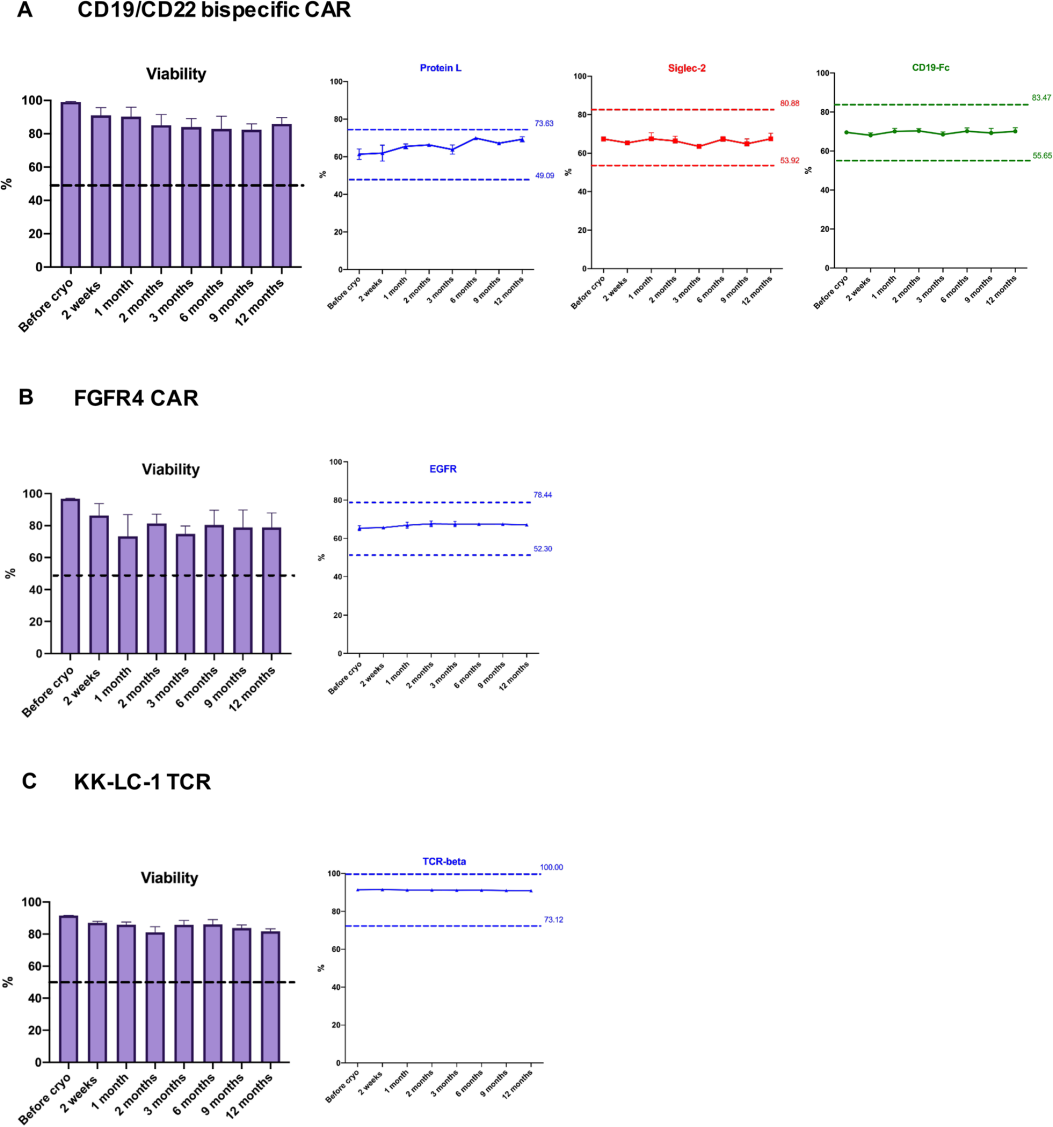
Evaluation of the long-term stability of the cryopreserved CAR/TCR T-cell flow cytometry quality controls (closed-vial stability). The cryopreserved flow cytometry quality control cells were thawed at 2 weeks, 1 month, 2 months, 3 months, 6 months, 9 months and 12 months and stained. The viability, CD3, CD4 and CD8 frequencies as well as surface CAR/TCR T-cell markers: protein L, CD22-Fc (Siglec-2) and CD19-Fc for CD19/CD22 bispecific CAR T-cell (**A**), EGFR for FGFR4 CAR-T cell (**B**) and murine TCR-beta for KK-LC-1 TCR T-cell (**C**) were measured by flow cytometry. Data are shown as mean ± SD. The upper and lower bounds (± 20% range of values measured at time of cryopreservation) are also shown in each plot

Cells were thawed at 2 weeks, 1 month, 2 months, 3 months, 6 months, 9 months and 12 months post-cryopreservation. Cell viability and CD3^+^, CD4^+^ and CD8^+^ T-cell frequencies as well as transduction efficiency and, for the bispecific CD19/CD22 CAR T-cells, identity were determined by flow cytometric analysis. As depicted in Fig. 2, the viability from all tested samples at each time point was over 50% measured by 7-AAD staining as well as by AO/PI viability staining (data not shown). We observed that the cryopreserved FGFR4 CAR T-cells and KK-LC-1 TCR T-cells expressed a similar level of EGFR or murine TCR-beta, respectively after thawing, but the transduction efficiency and the expression of identity markers on the CD19/CD22 bispecific T cell post-thaw slightly differed compared to the values measured at the time of cryopreservation. Nevertheless, the values from tested vials in all three products across the 12-month period remained within ± 20% of the values measured at the time of cryopreservation (Fig. 2A–C), showing that these cryopreserved cells are stable for at least one year. The mean, SD and CV of each parameter across the 12-month period as well as the passing criteria for each control cells were summarized in the Additional file 2: Table S2. Overall, our results demonstrated that the approach we used to establish a long-term quality control for the flow cytometry assay including cryopreservation and thawing of the CAR/TCR T-cells are valid, reliable and feasible.

### Determination of the shelf-life of thawed CAR/TCR T-cell flow cytometry quality control cells (open-vial stability)

**Fig. 3:**
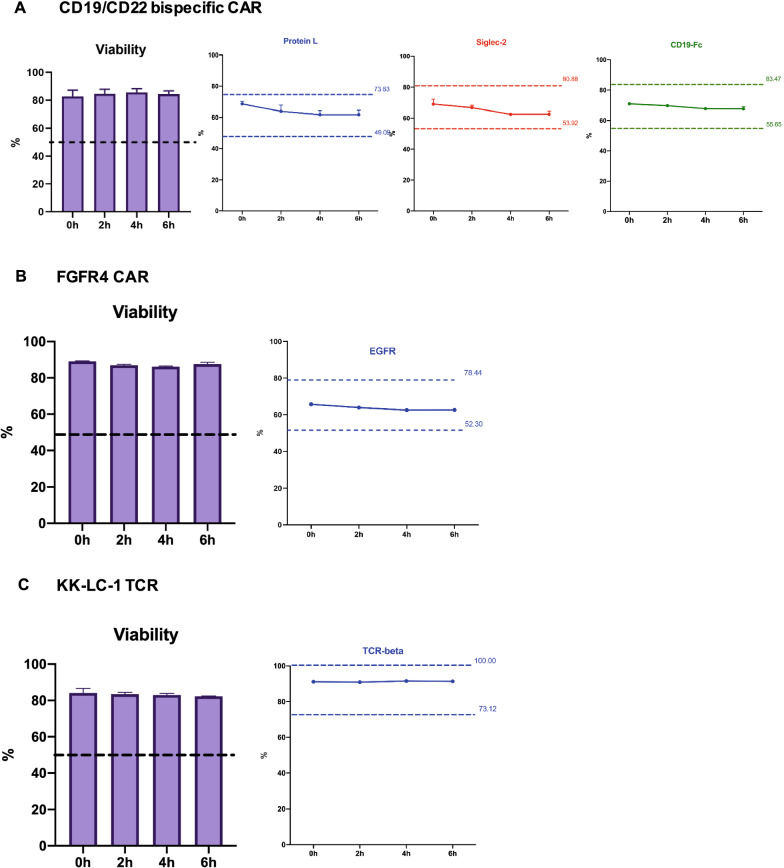
Determination of the shelf-life of the cryopreserved CAR/TCR T-cell flow cytometry quality controls post-thaw (open-vial stability). The cryopreserved flow cytometry quality control cells were thawed and stored at room temperature. The cells were stained at 0 h, 2 h, 4 h and 6 h post-thaw. Flow cytometry was used to assess cell viability and the expression of surface CAR/TCR markers: protein L, CD22-Fc (Siglec-2) and CD19-Fc for CD19/CD22 bispecific CAR-T cell (**A**), EGFR for FGFR4 CAR T cell (**B**) and murine TCR-beta for KK-LC-1 TCR T-cell (**C**). Data are shown as mean ± SD. The upper and lower bounds (± 20% range of values measured at time of cryopreservation) are also shown in each plot. Data are representative of two independent experiments with similar results. For CD19/CD22 bispecific CAR T-cell and KK-LC-1 TCR T-cell, the study was performed in triplicates. For FGFR4 CAR T-cell, the study was performed in duplicates

The evaluation of long-term stability of cryopreserved cells was addressed by studying the closed-vial stability. It is also important to determine the post-thaw shelf-life of cryopreserved CAR/TCR T-cells, which is known as open-vial stability. The three cryopreserved CAR/TCR T-cell quality controls were thawed as described above, stored at room temperature and evaluated by flow cytometry. The cells were stained at 0 h, 2 h, 4 h and 6 h post-thaw. Cell viability, transduction efficiency and proportion of cells expressing the identity marker were measured by flow cytometry. The viability of thawed cells in three cryopreserved quality controls did not fall over 6 h, indicating that cryopreserved CAR/TCR T-cells remained viable for at least 6 h post-thaw at room temperature (Fig. 3A–C). More importantly, the expression levels of the transduction efficiency and identity makers for three cryopreserved products were within ± 20% of the values measured at cryopreservation. All three types of control cells passed the criteria described above, although the values for two CAR-T control cell products were decreased slightly at the later time points compared to 0 h (Fig. 3A–C). The viability and expression levels of transduction efficiency and identity markers for all three quality control cells at each time point were summarized in the Additional file 2: Table S3. Thus, we concluded that cryopreserved CAR/TCR T-cells can be used for at least 6 h once a vial is thawed.

### Sensitivity of flow cytometry assays using the cryopreserved CAR/TCR T-cell flow cytometry quality control cells

**Fig. 4:**
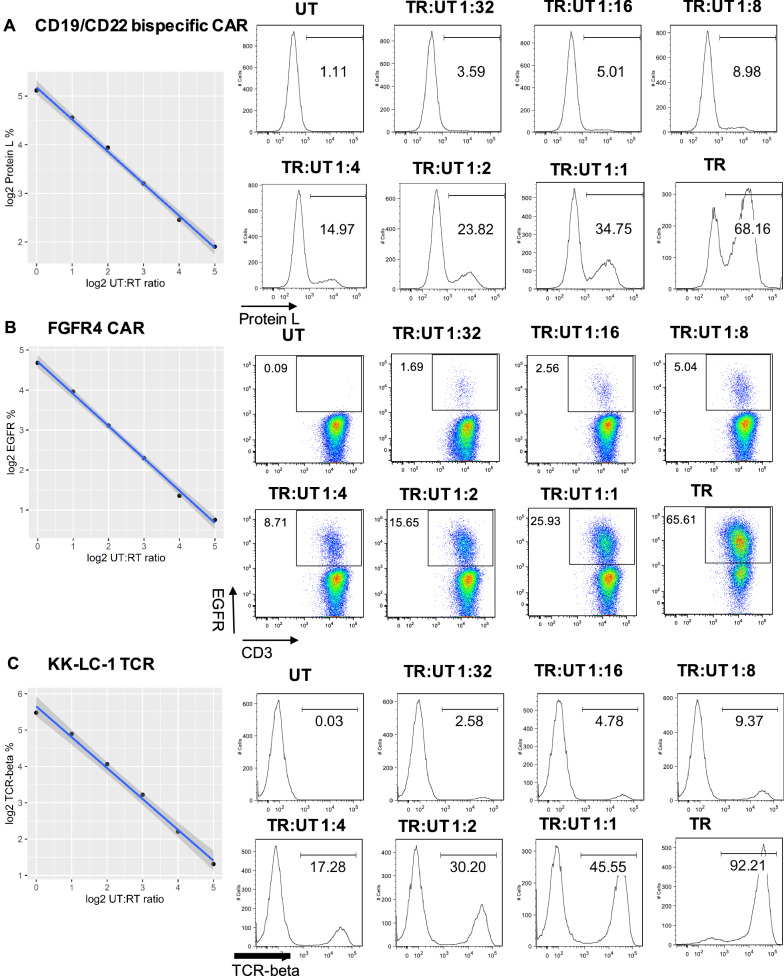
Examination of assays sensitivity when using the cryopreserved CAR/TCR T-cell flow cytometry quality controls. The cryopreserved flow cytometry quality control cells were thawed and diluted serially with the corresponding untransduced (UT) cells (1:1, 1:2, 1:4, 1:8, 1:16 and 1:32). The expression of transduction efficiency and identity markers: protein L, CD22-Fc (Siglec-2) and CD19-Fc for CD19/CD22 bispecific CAR-T cell (**A**), EGFR for FGFR4 CAR T-cell (**B**) and murine TCR-beta for KK-LC-1 TCR T-cell (**C**) were measured by flow cytometry. Data are shown as the correlation between the Log2 of the percentage of transduction efficiency and identity and the Log2 of dilution factor. The representative flow cytometry histograms or plots for protein L (**A**), EGFR (**B**) and TCR-beta (**C**) were gated on viable CD3^+^ cells. Data are representative of two independent experiments with similar results. For CD19/CD22 bispecific CAR T-cell, the study was performed in triplicates. For FGFR4 CAR T-cell and KK-LC-1 TCR T-cell, the study was performed in duplicates

To determine the dynamic detection range of the flow cytometry assay with cryopreserved CAR/TCR T-cell quality control cells, we mixed control cells with corresponding untransduced cells in serial dilutions. The lowest limit of quantification and linearity of expression of transduction efficiency and identity markers were determined in this study. As shown in Fig. 4A–C, the y axis was displayed as the Log2 of the percentages of transduction efficiency and identity markers, including protein L expression for CD19/CD22 bispecific CAR T-cells, EGFR expression for FGFR4 CAR T-cells as well as murine TCR-beta expression for KK-LC-1 TCR-T cells. The x axis was displayed as the Log2 of dilution factor. The Pearson correlation coefficient (r) were calculated using R package. There were strong associations between the expression level of transduction efficiency and dilution factors (r = − 0.9987 for CD19/CD22 bispecific CAR T-cells, r = − 0.9988 for FGFR4 CAR T-cells and r = − 0.9970 for KK-LC-1 TCR-T cells; p values were all less than 0.001). The lowest detection limit in our study was a 1:32 dilution. The mean and SD of transduction efficiency and identity markers for all three quality control cells at each dilution factor as well as r value and p value were summarized in the Additional file 2: Table S4. These results demonstrated that the flow cytometry assay using cryopreserved CAR/TCR T-cell controls is sensitive and reliable.

### Assessment of the precision of flow cytometry assays using the cryopreserved CAR/TCR T-cell flow cytometry quality control cells among assay laboratory staff

**Fig. 5:**
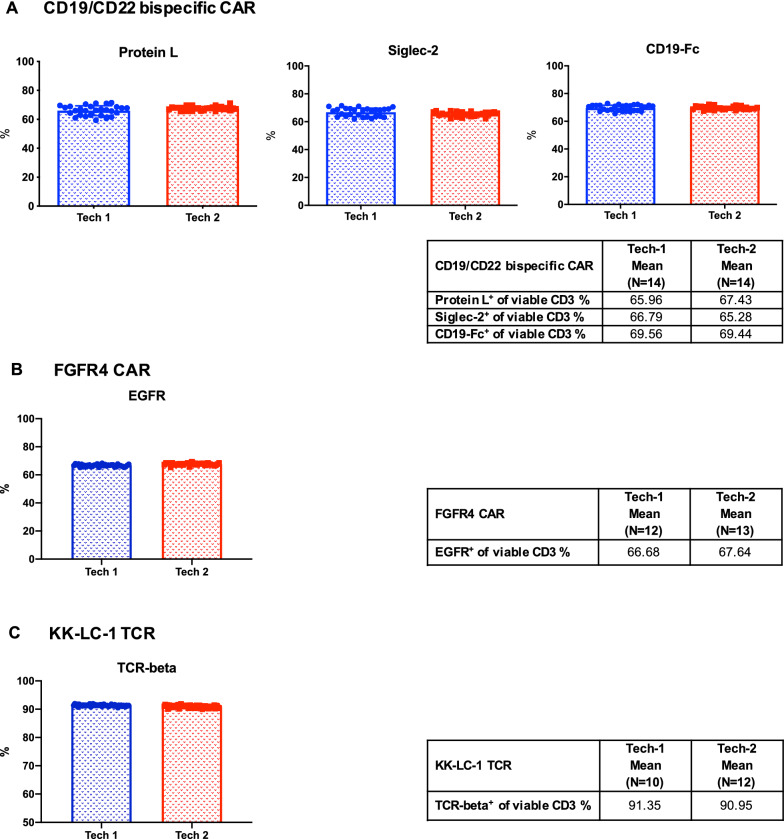
Assessment of assay precision among laboratory staff with the cryopreserved CAR/TCR T-cell flow cytometry quality controls. The cryopreserved flow cytometry quality control cells were thawed and stained by two laboratory technicians at all time points across the one-year long-term stability study as described above. The cell viability as well as the expression of the following CAR and TCR engineered T-cell surface markers: protein L, CD22-Fc (Siglec-2) and CD19-Fc for CD19/CD22 bispecific CAR T-cell (**A**), EGFR for FGFR4 CAR T-cell (**B**) and murine TCR-beta for KK-LC-1 TCR T-cell (**C**) were assessed by flow cytometry. Data are shown as mean ± SD. The mean values of transduction efficiency and identity markers of each quality control cells between Tech-1 and Tech-2 are summarized in each table

A critical step in the evaluation of quality control cells for flow cytometry is to determine if they generate consistent, reliable and accurate flow cytometry data among staff. Therefore, we first assessed the reproducibility of these cryopreserved control cells among different laboratory staff. Cryopreserved cells from each of the three CAR/TCR T-cell controls were thawed and stained by two lab technicians at all time points across one year. The viability and percentage of cells expressing the transduction efficiency and identity markers were determined by flow cytometry. The post-thaw viability measured by both technicians was over 50% (data not shown). Additionally, the percentage of transduction efficiency and identity markers for three CAR/TCR T-cell controls all passed ± 20% range criteria measured by both technicians (Fig. 5A–C). The results were summarized in the Additional file 2: Table S5 including the mean, SD and CV of each parameter between two technicians. In our study, when the CV was less than 5%, the values were considered to be comparable. Thus, these results demonstrated that flow cytometry assay results among technicians using these quality control cells are reproducible and reliable.

### Examination of the precision of flow cytometry assays using the cryopreserved CAR/TCR T-cell flow cytometry quality control cells across instruments

**Fig. 6:**
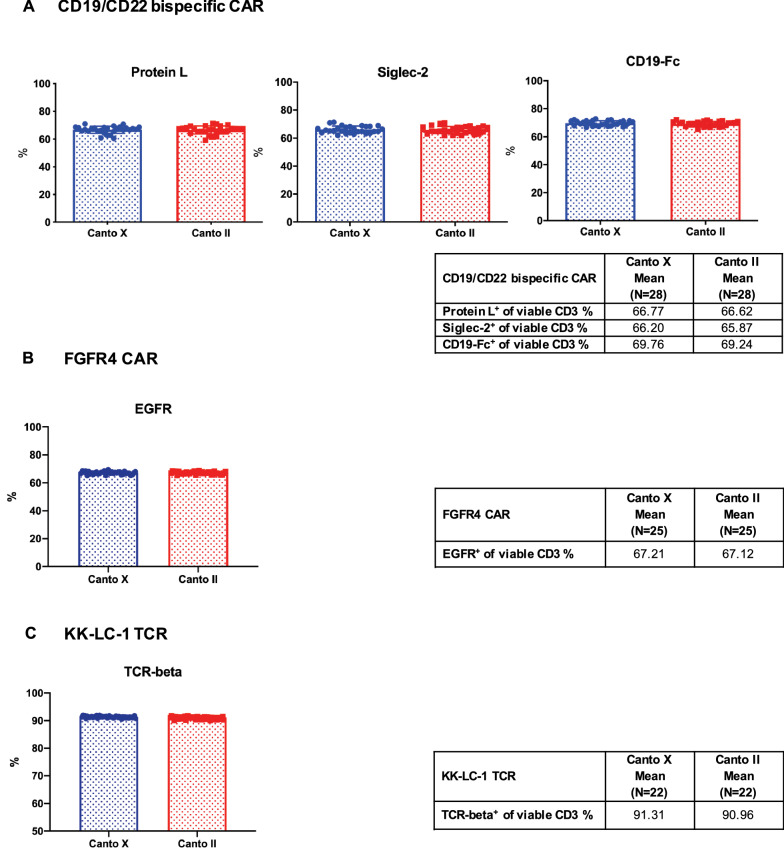
Examination of assay precision across instruments of cryopreserved CAR/TCR T-cell flow cytometry quality controls. The cryopreserved flow cytometry quality control cells were thawed, stained and measured on two flow instruments: BD FACSCanto II (Canto II) and BD FACSCanto 10-Color (Canto X) at all time points across the one-year long-term stability study as described above. The both instruments were used to assess cell viability as well as the expression of the following CAR and TCR engineered T-cell surface markers: protein L, CD22-Fc (Siglec-2) and CD19-Fc for CD19/CD22 bispecific CAR T-cell (**A**), EGFR for FGFR4 CAR T-cell (**B**) and murine TCR-beta for KK-LC-1 TCR T-cell (**C**). Data are shown as mean ± SD. The mean values of transduction efficiency and identity markers of each quality control cells between Canto II and Canto X are summarized in each table

In addition to comparing assay reproducibility among laboratory technicians, we also examined the performance of cryopreserved quality control cells on different instruments. The percentage of cells expressing the transduction efficiency and identity markers on each of the three cryopreserved CAR/TCR T-cell products was measured and compared using BD FACSCanto II (Canto II) and BD FACSCanto 10-Color (Canto X) instruments at all time points over 1 year. As shown in Fig. 6A–C, the percentage of cells expressing the transduction efficiency and identity markers was comparable between two instruments (CV from all tested parameters was less than 5%), demonstrating that the accuracy of flow cytometry assays using these cryopreserved control cells is not dependent on instruments type. The mean, SD and CV of each parameter between two instruments were listed in the Additional file 2: Table S6.

## Discussion

In this study, we performed a longitudinal study on cryopreserved genetically engineered T-cell flow cytometry quality controls obtained from two CAR-T cell products and one TCR engineered T-cell product. After thawing, these quality control cells provided a consistent, stable and accurate flow cytometry data across a 12-month period as well as among different laboratory staff and different instruments. We also showed that the cryopreserved quality control cells yielded sensitive flow cytometry assay results and that the cells were stable for 6 h after thawing. Thus, we developed a reliable and feasible procedure to establish an in-house cryopreserved CAR/TCR-T cell flow cytometry quality control and validated these cells.

Using a genetically engineered T-cell flow cytometry quality control to evaluate CAR T-cells and TCR-engineered T-cells during the manufacturing process is essential for maintaining compliance with cGMP regulations, especially for lot-release testing [14]. The quality control cells are thawed and stained along with patient samples. If values from quality control cells are within the established range and their viability remains over 50%, the assay is considered valid and the flow cytometry results from testing the clinical CAR T or TCR engineered T-cell product are accurate and the product can be released for fresh infusion or cryopreservation.

In this study, the passing criteria for our in-house CAR/TCR-T cell flow cytometry quality control included: (1) The percentage of cells expressing transduction efficiency and vector identity markers was within ± 20% range of values measured at time of cryopreservation; (2) The post-thaw cell viability was over 50% as measured by flow cytometric analysis via 7-AAD staining. These criteria were adopted from our institution’s established SOP for the post-thaw cells quality control. If cryopreserved control cells passed these two criteria after thawing, we considered the cells to be stable. We performed a longitudinal study one year in duration. Our results showed that the in-house cryopreserved control cells remained stable for at least 1 year, although the expression of some surface markers by thawed cells was lower than that of fresh cells. This may be due to the effects of cryopreservation and thawing procedure on CAR expression. However, one year of stability analysis is likely too short for a quality control product and we are continuing to examine the long-term stability of these cells.

We also investigated the post-thaw shelf-life of our in-house cryopreserved flow quality controls. We found that cryopreserved cells were stable for at least 6 h at room temperature after thawing. In our institution, the standard practice is to infuse cellular products within 4 h after thaw and storage at room temperature. Thus, we adopted the same post-thaw storage conditions for these in-house cryopreserved flow quality controls but extended the storage time to 6 h post-thaw. The cells may have a longer post-thaw shelf-life if they are kept on ice or at 4 degrees centigrade.

To determine the sensitivity of in-house cryopreserved CAR/TCR T-cell quality controls, we assessed the lowest limit of quantification and linearity of expression of transduction efficiency and vector identity markers by mixing control cells with corresponding untransduced cells in serial dilutions. There was a strong correlation between the expression of transduction efficiency and dilution factors, indicating that our in-house cryopreserved CAR/TCR T-cell controls are sensitive and reliable for flow cytometry assays. The lowest detection limit in our study was a 1:32 dilution. We may conduct further dilutions on FGFR4 CAR T-cell and KK-LC-1 TCR T-cell controls as we were still able to detect EGFR and TCR-beta expression at 1:32 dilution factor compared to the values from untransduced cells.

To evaluate the quality of our cryopreserved genetically engineered T-cell flow cytometry quality control, we conducted a precision assessment among laboratory staff. Although the percentages of some CAR T-cell markers measured by two staff differed slightly, the flow cytometry results were consistent (CV was less than 5% between two staff) and reliable. The parameters examined by two staff all passed the acceptance criteria. However, the study was performed only within our laboratory. If the cells are be used as a universal standard for multiple labs, inter-lab variation should be considered and evaluated in a future study.

We also evaluated the performance of cryopreserved positive cells on different instruments by measuring and comparing the transduction efficiency and vector identity on BD Canto II and BD Canto X instruments in our laboratory. BD Canto II can detect up to eight fluorescent markers with three lasers configuration. It is commonly used in the clinical flow cytometry laboratory. BD Canto X is a new model which can examine up to ten fluorochromes. The results showed that there was no significant difference between two BD flow cytometry instruments. However, both instruments belong to the same vendor and they share the same system and software for sample collection. Therefore, instruments from different vendors or different models from the same vendor should be included in the future validation plans.

Finally, we used two CAR T-cell products and one TCR-engineered T-cell product as examples for the establishment and validation of procedures for producing and using cryopreserved genetically engineered T-cells as a flow cytometry quality control. However, a wide variety of CAR T-cell and TCR-engineered T-cell products are being used in clinical trials and, in general, each type of CAR/TCR-T cell product requires a different marker for flow cytometry evaluation of transduction efficiency and identity testing and each engineered T-cell will require a unique quality control cell. While our study only provided a validation protocol to develop three specific cryopreserved CAR/TCR-T cell flow cytometry quality controls, for other CAR T-cell and TCR-engineered T-cell products, personalized protocols may need to be established.

## Conclusions

We developed a feasible and reliable procedure to establish and validate a cryopreserved CAR/TCR-T cell flow cytometry quality control which can serve as an assay quality control for in-process and lot-release testing of clinical CAR T-cell and TCR-engineered cell products.

## Supplementary Information

**Additional file 1: Figure S1. **The in-house cryopreserved CAR/TCR T-cell flow cytometry quality control validation schema. CAR/TCR-T cell products were manufactured in Center for Cellular Engineering (CCE), NIH Clinical Center. Cells were harvested at the end of culture and were cryopreserved and stored in liquid nitrogen. The validation procedure of cryopreserved CAR/TCR-T cell flow cytometry quality controls was shown in the schema, including: 1. The long-term stability (closed-vial stability); 2. The post-thaw shelf-life (open-vial stability); 3. The sensitivity assay; 4. The precision analysis among different technicians and different instruments. The passing criteria used in the study included: 1. The percentage of cells expressing transduction efficiency and vector identity markers was within ± 20% range of values measured at time of cryopreservation; 2. The post-thaw cell viability was over 50% as measured by flow cytometric analysis via 7-AAD staining. **Figure S2: **Gating strategy of Protein L expression on CD19/CD22 bispecific CAR-T product. Viable cells (7-AAD negative population) were first gated from the singlets of CD19/CD22 bispecific CAR transduced (TR) cells. CD3^+^ cells were then gated and analyzed for CD4^+^/CD8^+^ expression as well as protein L expression. The untransduced cells were stained at the same time and were used to identify the Protein L^+^ population on TR cells.

**Additional file 2.** **Table S1:** Summary of the characteristics of CD19/CD22 bispecific CAR T-cell /FGFR4 CAR T-cell /KK-LC-1 TCR T-cell flow cytometry quality control cells at cryopreservation. **Table S2:** Summary of the characteristics of CD19/CD22 bispecific CAR T-cell /FGFR4 CAR T-cell /KK-LC-1 TCR T-cell flow cytometry quality control cells post-thaw (long-term stability). **Table S3:** Summary of the characteristics of CD19/CD22 bispecific CAR T-cell /FGFR4 CAR T-cell /KK-LC-1 TCR T-cell flow cytometry quality control cells post-thaw at 0 hour, 2 hours, 4 hours and 6 hours (shelf-life). **Table S4:** Summary of the percentages of transduction efficiency and identity markers of CD19/CD22 bispecific CAR T-cell /FGFR4 CAR T-cell /KK-LC-1 TCR T-cell flow cytometry quality control cells post-thaw at each dilution factor. **Table S5:** Summary of the characteristics of CD19/CD22 bispecific CAR T-cell /FGFR4 CAR T-cell /KK-LC-1 TCR T-cell flow cytometry quality control cells post-thaw between two technicians. **Table S6:** Summary of the characteristics of CD19/CD22 bispecific CAR T-cell /FGFR4 CAR T-cell /KK-LC-1 TCR T-cell flow cytometry quality control cells post-thaw between two instruments

## Data Availability

Not applicable.
