# Supplementary material for: Establishment and validation of in-house cryopreserved CAR/TCR-T cell flow cytometry quality control
Source: J Transl Med. 2021 Dec 24;19:523. doi: 10.1186/s12967-021-03193-7 (PMC8705121; doi:10.1186/s12967-021-03193-7)

Supplemental Figure 1

The in-house cryopreserved CAR/TCR T-cell flow cytometry quality control validation schema

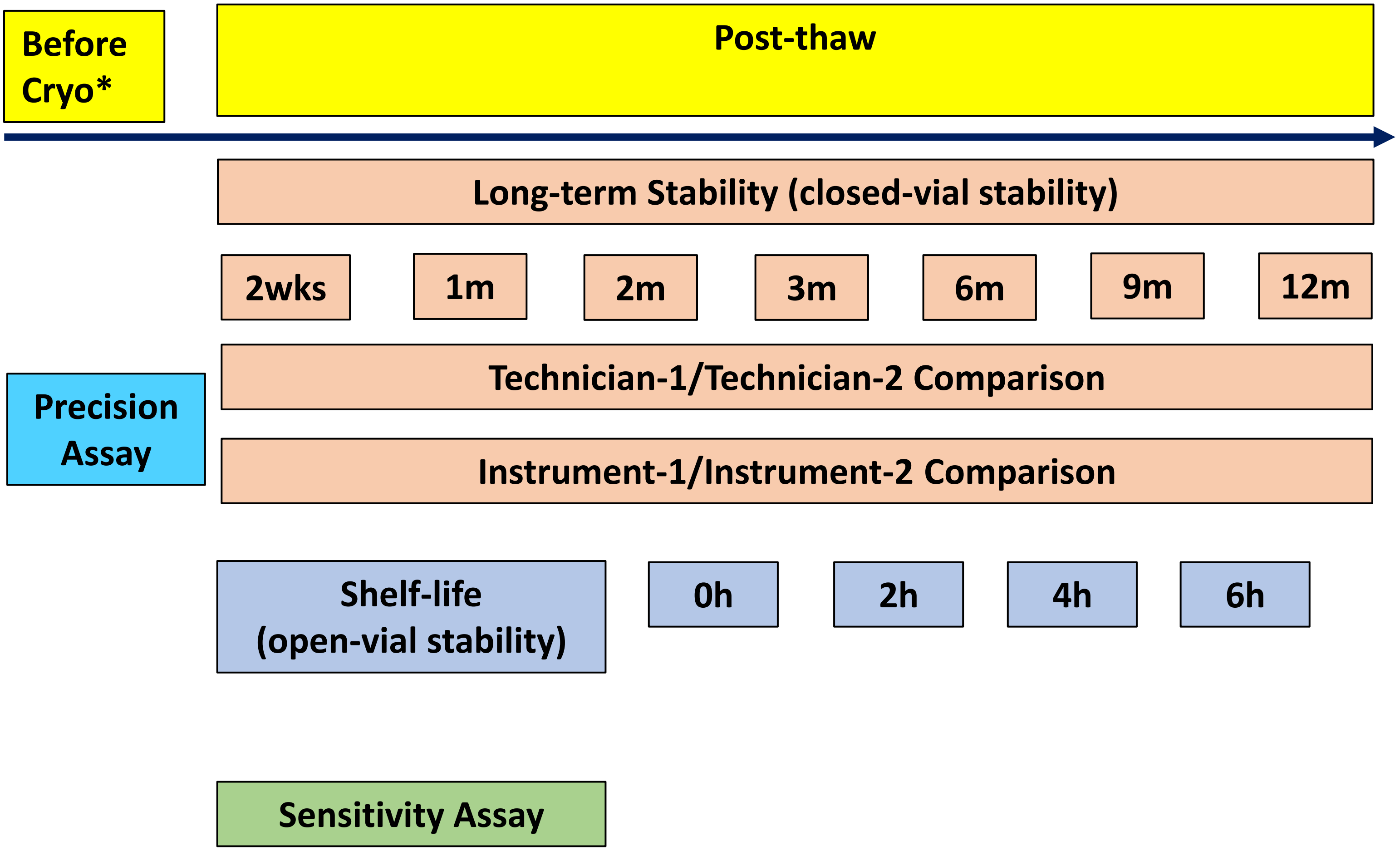

Passing Criteria

1. The percentage of cells expressing transduction efficiency and vector identity markers was within ± 20% range of values measured at time of cryopreservation.
2. The post-thaw cell viability was above 50% as measured by flow cytometric analysis via 7-AAD staining.

* Cryo: Cryopreservation

Supplemental Figure 2

Gating strategy of Protein L expression on fresh CD19/CD22 bispecific CAR-T cells

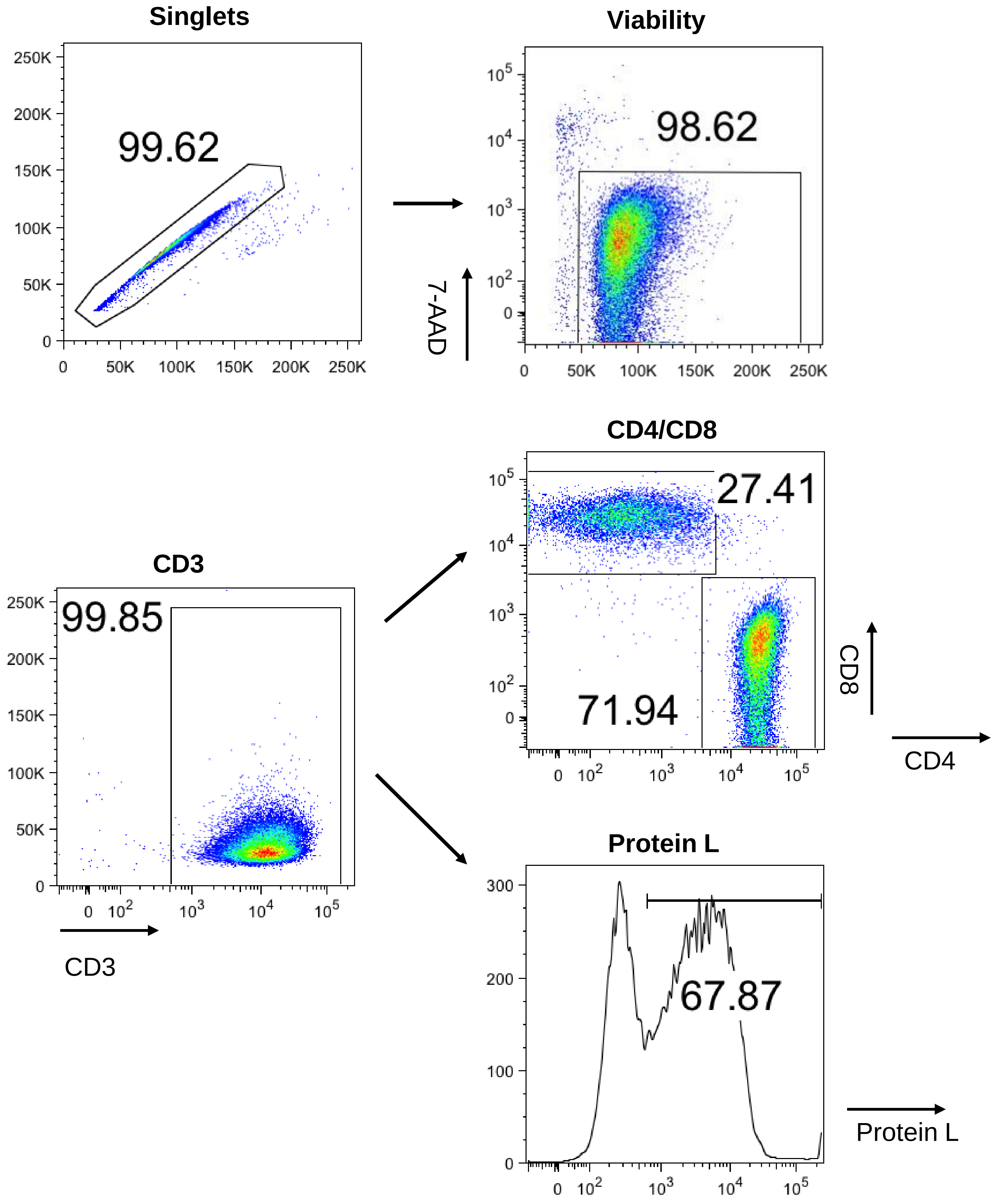

Supplement: Supplementary file 1 — Additional file 1: Figure S1. The in-house cryopreserved CAR/TCR T-cell flow cytometry quality control validation schema. CAR/TCR-T cell products were manufactured in Center for Cellular Engineering (CCE), NIH Clinical Center. Cells were harvested at the end of culture and were cryopreserved and stored in liquid nitrogen. The validation procedure of cryopreserved CAR/TCR-T cell flow cytometry quality controls was shown in the schema, including: 1. The long-term stability (closed-vial stability); 2. The post-thaw shelf-life (open-vial stability); 3. The sensitivity assay; 4. The precision analysis among different technicians and different instruments. The passing criteria used in the study included: 1. The percentage of cells expressing transduction efficiency and vector identity markers was within ± 20% range of values measured at time of cryopreservation; 2. The post-thaw cell viability was over 50% as measured by flow cytometric analysis via 7-AAD staining. Figure S2: Gating strategy of Protein L expression on CD19/CD22 bispecific CAR-T product. Viable cells (7-AAD negative population) were first gated from the singlets of CD19/CD22 bispecific CAR transduced (TR) cells. CD3+ cells were then gated and analyzed for CD4+/CD8+ expression as well as protein L expression. The untransduced cells were stained at the same time and were used to identify the Protein L+ population on TR cells. [file 12967_2021_3193_MOESM1_ESM.pdf]
