# Supplementary material for: Establishment and validation of in-house cryopreserved CAR/TCR-T cell flow cytometry quality control
Source: J Transl Med. 2021 Dec 24;19:523. doi: 10.1186/s12967-021-03193-7 (PMC8705121; doi:10.1186/s12967-021-03193-7)
Supplement: Supplementary file 2 — Additional file 2. Table S1: Summary of the characteristics of CD19/CD22 bispecific CAR T-cell /FGFR4 CAR T-cell /KK-LC-1 TCR T-cell flow cytometry quality control cells at cryopreservation. Table S2: Summary of the characteristics of CD19/CD22 bispecific CAR T-cell /FGFR4 CAR T-cell /KK-LC-1 TCR T-cell flow cytometry quality control cells post-thaw (long-term stability). Table S3: Summary of the characteristics of CD19/CD22 bispecific CAR T-cell /FGFR4 CAR T-cell /KK-LC-1 TCR T-cell flow cytometry quality control cells post-thaw at 0 hour, 2 hours, 4 hours and 6 hours (shelf-life). Table S4: Summary of the percentages of transduction efficiency and identity markers of CD19/CD22 bispecific CAR T-cell /FGFR4 CAR T-cell /KK-LC-1 TCR T-cell flow cytometry quality control cells post-thaw at each dilution factor. Table S5: Summary of the characteristics of CD19/CD22 bispecific CAR T-cell /FGFR4 CAR T-cell /KK-LC-1 TCR T-cell flow cytometry quality control cells post-thaw between two technicians. Table S6: Summary of the characteristics of CD19/CD22 bispecific CAR T-cell /FGFR4 CAR T-cell /KK-LC-1 TCR T-cell flow cytometry quality control cells post-thaw between two instruments [file 12967_2021_3193_MOESM2_ESM.pdf]

Supplemental Table 1

Summary of the Characteristics of CD19/CD22 bispecific CAR T-cell Flow Cytometry Quality Control Cells at Cryopreservation

	Mean	Standard Deviation (SD)	Coefficient of Variance (CV)
**Viability %**	99.08	0.27	0.27
**$CD3^+$ of viable %**	99.59	0.04	0.04
**$CD4^+$ of viable CD3 %**	71.50	0.46	0.65
**$CD8^+$ of viable CD3 %**	28.06	0.39	1.39
**Protein L^+ of viable CD3 %**	61.36	2.80	4.57
**Siglec-2^+ of viable CD3 %**	67.40	0.60	0.89
**CD19-Fc^+ of viable CD3 %**	69.56	0.46	0.66

Summary of the Characteristics of FGFR4 CAR T-cell Flow Cytometry Quality Control Cells at Cryopreservation

	Mean	Standard Deviation (SD)	Coefficient of Variance (CV)
**Viability %**	96.90	0.10	0.10
**$CD3^+$ of viable %**	99.95	0.01	0.01
**$CD4^+$ of viable CD3 %**	44.16	0.42	0.95
**$CD8^+$ of viable CD3 %**	54.51	0.31	0.57
**EGFR+ of viable CD3 %**	65.37	1.27	1.94

Summary of the Characteristics of KK-LC-1 TCR T-cell Flow Cytometry Quality Control Cells at Cryopreservation

	Mean	Standard Deviation (SD)	Coefficient of Variance (CV)
**Viability %**	91.57	0.21	0.23
**$CD3^+$ of viable %**	96.40	0.14	0.15
**$CD4^+$ of viable CD3 %**	64.51	0.60	0.93
**$CD8^+$ of viable CD3 %**	33.13	0.52	1.56
**TCR-$beta^+$ of viable CD3 %**	91.40	0.37	0.41

Supplemental Table 2

Summary of the Characteristics of CD19/CD22 bispecific CAR T-cell Flow Cytometry Quality Control Cells Post-thaw (long-term stability)

Post-thaw time	Parameters	Tested Sample Numbers	Mean	Standard Deviation (SD)	Coefficient of Variance (CV)	% of Samples Passing Criteria
2 weeks	Viability %	4	90.99	4.67	5.14	100 %
	$CD3^+$ of viable %		99.66	0.13	0.13	100 %
	$CD4^+$ of viable CD3 %		68.83	2.11	3.06	100 %
	$CD8^+$ of viable CD3 %		30.19	1.95	6.45	100 %
	Protein L^+ of viable CD3 %		61.93	4.21	6.80	100 %
	Siglec-2^+ of viable CD3 %		65.35	0.97	1.49	100 %
	CD19-Fc^+ of viable CD3 %		68.09	1.19	1.74	100 %
1 month	Viability %	4	90.23	5.63	6.24	100 %
	$CD3^+$ of viable %		99.78	0.07	0.07	100 %
	$CD4^+$ of viable CD3 %		69.06	1.68	2.43	100 %
	$CD8^+$ of viable CD3 %		30.28	1.73	5.70	100 %
	Protein L^+ of viable CD3 %		65.53	1.26	1.92	100 %
	Siglec-2^+ of viable CD3 %		67.48	3.16	4.68	100 %
	CD19-Fc^+ of viable CD3 %		70.04	1.54	2.19	100 %
2 months	Viability %	4	85.12	6.30	7.40	100 %
	$CD3^+$ of viable %		99.75	0.21	0.21	100 %
	$CD4^+$ of viable CD3 %		67.08	2.41	3.59	100 %
	$CD8^+$ of viable CD3 %		32.18	2.44	7.57	100 %
	Protein L^+ of viable CD3 %		66.28	0.87	1.32	100 %
	Siglec-2^+ of viable CD3 %		66.34	2.47	3.72	100 %
	CD19-Fc^+ of viable CD3 %		70.34	1.15	1.64	100 %
3 months	Viability %	4	83.96	5.04	6.01	100 %
	$CD3^+$ of viable %		98.95	0.95	0.96	100 %
	$CD4^+$ of viable CD3 %		66.40	1.78	2.69	100 %
	$CD8^+$ of viable CD3 %		32.83	1.70	5.17	100 %
	Protein L^+ of viable CD3 %		63.80	2.45	3.84	100 %
	Siglec-2^+ of viable CD3 %		63.50	1.03	1.62	100 %
	CD19-Fc^+ of viable CD3 %		68.47	1.30	1.91	100 %
6 months	Viability %	4	82.86	7.59	9.16	100 %
	$CD3^+$ of viable %		96.17	3.02	3.14	100 %
	$CD4^+$ of viable CD3 %		69.41	2.00	2.89	100 %
	$CD8^+$ of viable CD3 %		29.98	1.90	6.35	100 %
	Protein L^+ of viable CD3 %		69.87	0.80	1.15	100 %
	Siglec-2^+ of viable CD3 %		67.23	1.34	2.00	100 %
	CD19-Fc^+ of viable CD3 %		70.22	1.62	2.31	100 %
9 months	Viability %	4	82.41	3.55	4.30	100 %
	$CD3^+$ of viable %		99.69	0.32	0.32	100 %
	$CD4^+$ of viable CD3 %		67.90	0.78	1.15	100 %
	$CD8^+$ of viable CD3 %		31.43	0.73	2.31	100 %
	Protein L^+ of viable CD3 %		67.20	0.55	0.82	100 %
	Siglec-2^+ of viable CD3 %		64.87	2.55	3.94	100 %
	CD19-Fc^+ of viable CD3 %		69.24	2.33	3.37	100 %
12 months	Viability %	4	85.83	3.75	4.37	100 %
	$CD3^+$ of viable %		99.65	0.30	0.30	100 %
	$CD4^+$ of viable CD3 %		67.68	1.37	2.03	100 %
	$CD8^+$ of viable CD3 %		31.64	1.28	4.03	100 %
	Protein L^+ of viable CD3 %		67.29	1.36	1.96	100 %
	Siglec-2^+ of viable CD3 %		57.48	2.90	4.30	100 %
	CD19-Fc^+ of viable CD3 %		70.11	1.85	2.63	100 %

Passing Criteria:

1. Viability (7-AAD negative population) ≥ 50 %
2. $CD3^+$ of viable %: 79.67 – 100.00 %
3. $CD4^+$ of viable CD3 %: 57.20 – 85.80 %
4. $CD8^+$ of viable CD3 %: 22.45 – 33.67 %
5. Protein L^+ of viable CD3 %: 49.09 – 73.63 %
6. Siglec-2^+ of viable CD3 %: 53.92 – 80.88 %
7. CD19-Fc^+ of viable CD3 %: 55.65 – 83.47 %

Summary of the Characteristics of FGFR4 CAR T-cell Flow Cytometry Quality Control Cells Post-thaw (long-term stability)

Post-thaw Time	Parameters	Tested Sample Numbers	Mean	Standard Deviation (SD)	Coefficient of Variance (CV)	% of Samples Passing Criteria
2 weeks	**Viability %**	3	86.32	7.51	8.70	100 %
	**$CD3^+$ of viable %**		99.94	0.02	0.02	100 %
	**$CD4^+$ of viable CD3 %**		41.24	1.34	1.39	100 %
	**$CD8^+$ of viable CD3 %**		54.46	1.39	2.56	100 %
	**$EGFR^+$ of viable CD3 %**		65.66	0.48	0.74	100 %
1 month	**Viability %**	3	73.12	13.75	18.80	100 %
	**$CD3^+$ of viable %**		99.96	0.02	0.02	100 %
	**$CD4^+$ of viable CD3 %**		44.01	1.76	4.01	100 %
	**$CD8^+$ of viable CD3 %**		52.88	3.11	5.89	100 %
	**$EGFR^+$ of viable CD3 %**		66.90	1.55	2.32	100 %
2 months	**Viability %**	3	81.33	5.76	7.08	100 %
	**$CD3^+$ of viable %**		99.95	0.02	0.02	100 %
	**$CD4^+$ of viable CD3 %**		43.96	2.84	6.46	100 %
	**$CD8^+$ of viable CD3 %**		53.23	3.41	6.41	100 %
	**$EGFR^+$ of viable CD3 %**		67.63	1.45	2.15	100 %
3 months	**Viability %**	3	75.93	3.81	5.02	100 %
	**$CD3^+$ of viable %**		99.96	0.01	0.01	100 %
	**$CD4^+$ of viable CD3 %**		43.33	2.61	6.02	100 %
	**$CD8^+$ of viable CD3 %**		53.54	3.40	6.35	100 %
	**$EGFR^+$ of viable CD3 %**		67.51	1.35	2.00	100 %
6 months	**Viability %**	4	80.28	9.33	11.63	100 %
	**$CD3^+$ of viable %**		99.98	0.02	0.02	100 %
	**$CD4^+$ of viable CD3 %**		43.91	1.94	4.42	100 %
	**$CD8^+$ of viable CD3 %**		53.99	2.49	4.61	100 %
	**$EGFR^+$ of viable CD3 %**		67.50	0.63	0.93	100 %
9 months	**Viability %**	4	78.97	11.16	14.13	100 %
	**$CD3^+$ of viable %**		99.97	0.03	0.03	100 %
	**$CD4^+$ of viable CD3 %**		42.04	1.97	4.69	100 %
	**$CD8^+$ of viable CD3 %**		56.01	2.04	3.65	100 %
	**$EGFR^+$ of viable CD3 %**		67.35	0.40	0.59	100 %
12 months	**Viability %**	4	78.78	9.15	11.61	100 %
	**$CD3^+$ of viable %**		99.95	0.05	0.05	100 %
	**$CD4^+$ of viable CD3 %**		43.36	3.15	7.27	100 %
	**$CD8^+$ of viable CD3 %**		54.97	3.10	5.65	100 %
	**$EGFR^+$ of viable CD3 %**		67.10	0.44	0.66	100 %

Passing Criteria:

1. Viability (7-AAD negative population) ≥ 50 %
2. $CD3^+$ of viable %: 79.96 – 100.00 %
3. $CD4^+$ of viable CD3 %: 35.33 – 52.99 %
4. $CD8^+$ of viable CD3 %: 43.61 – 65.41 %
5. $EGFR^+$ of viable CD3 %: 52.30 – 78.44 %

Summary of the Characteristics of KK-LC-1 TCR T-cell Flow Cytometry Quality Control Cells Post-thaw (long-term stability)

Post-thaw Time	Parameters	Tested Sample Numbers	Mean	Standard Deviation (SD)	Coefficient of Variance (CV)	% of Samples Passing Criteria
2 weeks	Viability %	3	87.01	0.92	1.05	100 %
	$CD3^+$ of viable %		96.43	0.33	0.35	100 %
	$CD4^+$ of viable CD3 %		63.86	0.80	1.25	100 %
	$CD8^+$ of viable CD3 %		33.82	0.68	2.02	100 %
	TCR-$beta^+$ of viable CD3 %		91.51	0.31	0.34	100 %
1 month	Viability %	3	85.88	1.67	1.94	100 %
	$CD3^+$ of viable %		96.39	0.64	0.66	100 %
	$CD4^+$ of viable CD3 %		63.16	0.84	1.33	100 %
	$CD8^+$ of viable CD3 %		34.30	0.59	1.72	100 %
	TCR-$beta^+$ of viable CD3 %		91.31	0.45	0.49	100 %
2 months	Viability %	3	81.11	3.55	4.38	100 %
	$CD3^+$ of viable %		96.74	0.36	0.37	100 %
	$CD4^+$ of viable CD3 %		62.97	0.81	1.28	100 %
	$CD8^+$ of viable CD3 %		34.27	0.61	1.79	100 %
	TCR-$beta^+$ of viable CD3 %		91.31	0.43	0.47	100 %
3 months	Viability %	3	85.77	2.81	3.27	100 %
	$CD3^+$ of viable %		96.76	0.40	0.41	100 %
	$CD4^+$ of viable CD3 %		63.57	1.22	1.92	100 %
	$CD8^+$ of viable CD3 %		33.46	0.92	2.74	100 %
	TCR-$beta^+$ of viable CD3 %		91.17	0.50	0.55	100 %
6 months	Viability %	3	86.03	2.99	3.48	100 %
	$CD3^+$ of viable %		96.95	0.33	0.34	100 %
	$CD4^+$ of viable CD3 %		63.14	1.05	1.67	100 %
	$CD8^+$ of viable CD3 %		34.03	0.81	2.38	100 %
	TCR-$beta^+$ of viable CD3 %		91.18	0.23	0.26	100 %
9 months	Viability %	4	83.83	1.92	2.29	100 %
	$CD3^+$ of viable %		96.93	0.35	0.36	100 %
	$CD4^+$ of viable CD3 %		63.38	1.03	1.62	100 %
	$CD8^+$ of viable CD3 %		33.53	0.97	2.88	100 %
	TCR-$beta^+$ of viable CD3 %		91.02	0.37	0.40	100 %
12 months	Viability %	4	81.70	1.60	1.96	100 %
	$CD3^+$ of viable %		96.69	0.48	0.50	100 %
	$CD4^+$ of viable CD3 %		62.87	0.71	1.12	100 %
	$CD8^+$ of viable CD3 %		34.16	0.50	1.46	100 %
	TCR-$beta^+$ of viable CD3 %		90.97	0.50	0.55	100 %

Passing Criteria:

1. Viability (7-AAD negative population) ≥ 50 %
2. $CD3^+$ of viable %: 77.12 – 100.00 %
3. $CD4^+$ of viable CD3 %: 51.61 – 77.41 %
4. $CD8^+$ of viable CD3 %: 26.50 – 39.76 %
5. TCR-$beta^+$ of viable CD3 %: 73.12 – 100.00 %

Supplemental Table 3

Summary of the Characteristics of CD19/CD22 bispecific CAR T-cell Flow Cytometry Quality Control Cells Post-thaw at 0 hour, 2 hours, 4 hours and 6 hours (shelf-life)

Post-thaw Time	Parameters	Mean	Standard Deviation (SD)	Coefficient of Variance (CV)
0 hour	Viability %	82.78	4.51	5.44
	Protein L^{+} of viable CD3 %	68.65	1.66	2.41
	Siglec-2^{+} of viable CD3 %	69.08	3.13	4.53
	CD19-Fc^{+} of viable CD3 %	70.95	0.51	0.71
2 hours	Viability %	84.55	3.36	3.97
	Protein L^{+} of viable CD3 %	63.87	4.08	6.39
	Siglec-2^{+} of viable CD3 %	66.81	1.57	2.34
	CD19-Fc^{+} of viable CD3 %	69.73	0.59	0.84
4 hours	Viability %	85.64	2.78	3.24
	Protein L^{+} of viable CD3 %	61.75	3.06	4.96
	Siglec-2^{+} of viable CD3 %	62.48	0.90	1.45
	CD19-Fc^{+} of viable CD3 %	67.78	0.57	0.83
6 hours	Viability %	84.46	2.55	3.02
	Protein L^{+} of viable CD3 %	61.73	3.52	5.71
	Siglec-2^{+} of viable CD3 %	62.50	2.14	3.42
	CD19-Fc^{+} of viable CD3 %	67.70	1.36	2.01

Summary of the Characteristics of FGFR4 CAR T-cell Flow Cytometry Quality Control Cells Post-thaw at 0 hour, 2 hours, 4 hours and 6 hours (shelf-life)

Post-thaw Time	Parameters	Mean	Standard Deviation (SD)	Coefficient of Variance (CV)
0 hour	Viability %	89.09	0.29	0.33
	EGFR^{+} of viable CD3 %	65.75	0.57	0.86
2 hours	Viability %	86.95	0.47	0.54
	EGFR^{+} of viable CD3 %	63.92	0.64	1.01
4 hours	Viability %	86.20	0.36	0.42
	EGFR^{+} of viable CD3 %	62.55	0.69	1.11
6 hours	Viability %	87.62	1.03	1.18
	EGFR^{+} of viable CD3 %	62.62	0.80	1.28

Summary of the Characteristics of KK-LC-1 TCR T-cell Flow Cytometry Quality Control Cells Post-thaw at 0 hour, 2 hours, 4 hours and 6 hours (shelf-life)

Post-thaw Time	Parameters	Mean	Standard Deviation (SD)	Coefficient of Variance (CV)
0 hour	Viability %	84.11	2.49	2.96
	TCR-beta^{+} of viable CD3 %	91.13	0.09	0.10
2 hours	Viability %	83.43	0.99	1.18
	TCR-beta^{+} of viable CD3 %	90.89	0.07	0.08
4 hours	Viability %	83.04	0.83	1.00
	TCR-beta^{+} of viable CD3 %	91.52	0.05	0.06
6 hours	Viability %	82.25	0.17	0.20
	TCR-beta^{+} of viable CD3 %	91.35	0.27	0.29

Supplemental Table 4

Summary of the Percentages of Transduction Efficiency and Identity Markers of CD19/CD22 bispecific CAR T-cell /FGFR4 CAR T-cell /KK-LC-1 TCR T-cell Flow Cytometry Quality Control Cells Post-thaw at Each Dilution Factor

	CD19/CD22 bispecific CAR T-cell Protein L$^+$ of viable CD3 % Mean ± SD	FGFR4 CAR T-cell EGFR$^+$ of viable CD3 % Mean ± SD	KK-LC-1 TCR T-cell TCR-beta$^+$ of viable CD3 % Mean ± SD
**UT**	1.73 ± 0.76	0.09 ± 0.01	0.03 ± 0.01
**TR: UT 1:32**	3.75 ± 0.22	1.70 ± 0.01	2.52 ± 0.08
**TR: UT 1:16**	5.49 ± 0.53	2.59 ± 0.04	4.69 ± 0.13
**TR: UT 1:8**	9.21 ± 0.23	5.00 ± 0.06	9.40 ± 0.04
**TR: UT 1:4**	15.35 ± 0.15	8.80 ± 0.12	16.94 ± 0.48
**TR: UT 1:2**	23.61 ± 0.29	15.71 ± 0.08	30.11 ± 0.13
**TR: UT 1:1**	34.60 ± 1.19	25.84 ± 0.13	44.93 ± 0.87
**TR**	67.63 ± 0.48	65.94 ± 0.45	92.13 ± 0.13
**Pearson correlation coefficient (r)**	-0.9987	-0.9988	-0.9970
**p value**	<0.001	<0.001	<0.001

Supplemental Table 5

Summary of the Characteristics of CD19/CD22 bispecific CAR T-cell /FGFR4 CAR T-cell /KK-LC-1 TCR T-cell Flow Cytometry Quality Control Cells Post-thaw Between Two Technicians

Cryopreserved CAR/TCR T-Cell Quality Controls	Transduction Efficiency and Identity Markers	Tech-1 Mean	Tech-2 Mean	Standard Deviation (SD)	Coefficient of Variance (CV)
**CD19/CD22 bispecific CAR T-cell**	**Protein L$^+$ of viable CD3 %**	65.96	67.43	1.04	1.56
	**Siglec-2$^+$ of viable CD3 %**	66.79	65.28	1.07	1.62
	**CD19-Fc$^+$ of viable CD3 %**	69.56	69.44	0.08	0.12
**FGFR4 CAR T-cell**	**EGFR$^+$ of viable CD3 %**	66.68	67.64	0.68	1.01
**KK-LC-1 TCR T-cell**	**TCR-beta$^+$ of viable CD3 %**	91.35	90.95	0.28	0.31

Tested sample numbers:
CD19/CD22 bispecific CAR T-cell: Tech-1: 14; Tech-2: 14
FGFR4 CAR T-cell: Tech-1: 12; Tech-2: 13
KK-LC-1 TCR T-cell: Tech-1: 10; Tech-2: 12

Supplemental Table 6

Summary of the Characteristics of CD19/CD22 bispecific CAR T-cell /FGFR4 CAR T-cell /KK-LC-1 TCR T-cell Flow Cytometry Quality Control Cells Post-thaw Between Two Instruments

Cryopreserved CAR/TCR T-Cell Quality Controls	Transduction Efficiency and Identity Markers	Canto X Mean	Canto II Mean	Standard Deviation (SD)	Coefficient of Variance (CV)
CD19/CD22 bispecific CAR T-cell	Protein L^{+} of viable CD3 %	66.77	66.62	0.10	0.15
	Siglec-2^{+} of viable CD3 %	66.20	65.87	0.23	0.35
	CD19-Fc^{+} of viable CD3 %	69.76	69.24	0.37	0.53
FGFR4 CAR T-cell	EGFR^{+} of viable CD3 %	67.21	67.12	0.06	0.09
KK-LC-1 TCR T-cell	TCR-beta^{+} of viable CD3 %	91.31	90.96	0.25	0.27

Tested sample numbers:
CD19/CD22 bispecific CAR T-cell: Canto II: 28; Canto X: 28
FGFR4 CAR T-cell: Canto II: 25; Canto X: 25
KK-LC-1 TCR T-cell: Canto II: 22; Canto X: 22
